# Thank You

**Published:** 1914-10

**Authors:** 


### Thank You.

So many of the JOURNAL'S friends have paid many years (some to 1920) in advance, that I am going to take this opportunity to thank you all at once.

It does look good to receive a five dollar bill and have some doctor write, "please credit my subscription to the amount of five dollars." In fact, no editor ever appreciated such a mark of kindness and thoughtfulness quite so much as do I, because it is a delicate way of saying, "we have confidence in your ability to give us a good journal and we are going to give you our help and encouragement." I do thank every one of you from the depths of my heart, and assure you that nothing inspires me to give you the best of everything like this mark of your confidence in me and your appreciation of my efforts to make the JOURNAL helpful and useful to the profession, as manifested in this substantial support.

